# The Regulatory Role of Rac1, a Small Molecular Weight GTPase, in the Development of Diabetic Retinopathy

**DOI:** 10.3390/jcm8070965

**Published:** 2019-07-03

**Authors:** Nikhil Sahajpal, Anjan Kowluru, Renu A. Kowluru

**Affiliations:** 1 Department of Ophthalmology, Visual and Anatomical Sciences, Detroit, MI, 48201, USA; 2 Pharmaceutical Sciences, Wayne State University, Detroit, MI, 48201, USA; 3 Wayne State University, and John D Dingell VA Medical Center, Detroit, MI, 48201, USA

**Keywords:** diabetic retinopathy, epigenetics, oxidative stress, Rac1

## Abstract

Diabetic retinopathy, a microvascular complication of diabetes, remains the leading cause of vision loss in working age adults. Hyperglycemia is considered as the main instigator for its development, around which other molecular pathways orchestrate. Of these multiple pathways, oxidative stress induces many metabolic, functional and structural changes in the retinal cells, leading to the development of pathological features characteristic of this blinding disease. An increase in cytosolic reactive oxygen species (ROS), produced by cytosolic NADPH oxidase 2 (Nox2), is an early event in the pathogenesis of diabetic retinopathy, which leads to mitochondrial damage and retinal capillary cell apoptosis. Activation of Nox2 is mediated through an obligatory small molecular weight GTPase, Ras-related C3 botulinum toxin substrate 1 (Rac1), and subcellular localization of Rac1 and its activation are regulated by several regulators, rendering it a complex biological process. In diabetes, Rac1 is functionally activated in the retina and its vasculature, and, via Nox2-ROS, contributes to mitochondrial damage and the development of retinopathy. In addition, *Rac1* is also transcriptionally activated, and epigenetic modifications play a major role in this transcriptional activation. This review focusses on the role of Rac1 and its regulation in the development and progression of diabetic retinopathy, and discusses some possible avenues for therapeutic interventions.

## 1. Introduction

Diabetes has become a major public health burden, and is now considered to be an epidemic in the 21st century. In 2015, diabetes affected 415 million people globally, and these numbers are expected to reach 642 million by 2040 (International Diabetes Federation. IDF Diabetes Atlas, 7th Edition). Diabetes leads to structural and functional changes in both the macro- and micro-vasculature of tissue; the macrovascular alterations are observed mainly in cardiovascular, cerebrovascular and peripheral artery diseases, and the microvascular alterations result in retinopathy, nephropathy and neuropathy [1]. Though hyperglycemia has been implicated as a causal link between diabetes and its complications [2,3,4], a clear understanding of the mechanism(s) of the development of these complications remain complex as the genetic and environmental factors also orchestrate these mechanism(s) to varied degrees.

Chronic hyperglycemia damages the retina, leading to progressive vision loss and ultimately blindness. It is the leading cause of blindness in working age adults around the world, and its incidence is expected to increase owing to the increasing global diabetes burden each year [5]. The global prevalence of diabetic retinopathy was about 126.6 million in 2010, and this number is expected to escalate to 191 million by 2030 [6]. In about one third of diabetic patients, retinopathy progresses to vision threatening stage, and 2% of the patients become blind. The etiology of diabetic retinopathy follows a distinctive trend, where one feature succumbs to another as the disease progresses; while the early clinical signs are microaneurysms, small hemorrhages and leakage of lipids into the retina, but with time this progresses to capillary closure and retinal hypoxia [1,7]. Inevitably, excessive capillary closure leads to abnormal proliferation of retinal vessels, accompanied by vitreous hemorrhage and fibrosis resulting in proliferative diabetic retinopathy [8].

Several modifiable risk factors such as hyperglycemia, hypertension, dyslipidemia and obesity, have been associated with diabetic retinopathy, but the significance of hyperglycemia transcends any other factor [9]. The Diabetes Control and Complications Trial (DCCT) has clearly documented a close association between tight glycemic control (HbA1c < 7%) and reduced risk of the development/progression of diabetic retinopathy in type 1 diabetic patients. The incidence of retinopathy is reduced by 76%, and that of progression to advanced retinopathy, by 54%, if the intensive glycemic control is maintained [2].The United Kingdom Prospective Diabetes Study also documented similar association between the severity of hyperglycemia and the incidence of diabetic retinopathy in type 2 diabetic patients [4]. However, maintenance of intensive control is difficult for this life-long disease, which requires modification of life style, dedication by the patient and the loved ones, and carries an increased risk of hypoglycemic seizure and possible weight gain [10]. Thus, understanding the molecular mechanism of its development is essential for identifying potential future therapies for retinopathy in diabetes.

## 2. Molecular Mechanisms of Diabetic Retinopathy

The extensive research conducted over the years has shed enormous light on the various molecular players and pathways that orchestrate the development and progression of diabetic retinopathy. The persistent hyperglycemia is considered as the primary causative factor that leads to its development. Activation of polyol pathway, protein kinase C, increased oxidative stress and advanced glycation product formation are some of the pathways that are considered to play a role in the development diabetic retinopathy. Of these multiple pathways, oxidative stress is considered to be one of the major pathways in the complex pathophysiological of diabetic retinopathy [11,12,13].

Hyperglycemia can generate reactive oxygen species (ROS) via autoxidizing glucose or increasing cytosolic NADPH oxidase (Nox) activity, or by affecting mitochondrial respiratory chain, and ROS damage biomolecules including DNA, lipids, proteins and carbohydrates [11,14,15]. Experimental models have shown that an increase in cytosolic ROS, produced by Nox2, is an early event in the pathogenesis of diabetic retinopathy, which precedes mitochondrial damage, further increasing free radicals [15]. Continuous ROS production damages mitochondrial DNA (mtDNA), damaged mtDNA further compromises the electron transport chain by reducing the transcription of mtDNA-encoded genes critical for its functioning, and the vicious cycle of free radicals continues to self-propagate. Damage to mitochondrial membranes by ROS renders them permeable, releasing cytochrome C into the cytoplasm. This serves as a signal of cell apoptosis, and capillary cells begin to undergo accelerated apoptosis [16,17]. Persistent hyperglycemia, excessive free radicals and dysfunctional mitochondria form a conundrum that further propagates these events, leading to cell apoptosis and the development and progression of diabetic retinopathy. 

## 3. NADPH Oxidases

The cytosolic Nox is the primary enzyme responsible for the generation of cellular ROS [18]. The Nox family comprises of seven different isoforms, each with multiple large transmembrane catalytic subunits, catalyzing the reduction of molecular oxygen to superoxide anion by oxidizing cytosolic NADPH to NADP. The seven Nox genes have been categorized as Nox1, Nox2, Nox3, Nox4, Nox5, Duox1 and Duox 2 [19]. Of these isoforms, Nox2 is present in numerous cell types, including retina. Though, initially regarded as a phagocytic enzyme, its altered activity in non-phagocytic cells has been associated with the development of several pathologies including beta cell loss [20]. In addition to Nox2, other members of the Nox family, specifically Nox1 and Nox4, are also involved in cellular dysfunction leading to pathological conditions, and altered activities of Nox1, Nox2 and Nox4 are seen in specific retinal cell types including endothelial cells and pericytes, in diabetes, implicating them in the development and progression of diabetic retinopathy [15,21,22]. Moreover, a recent genome wide association study has demonstrated the association of the Nox4 gene with severe diabetic retinopathy in type 2 diabetes patients [23]. The present review focuses on Rac1-Nox2, and the role of other isoforms of Nox in diabetic retinopathy is beyond the scope of this review. 

Nox2 is constitutively associated with p22^phox^ on the membrane but requires a complex process of protein-protein interactions for its activation. The p47^phos^ subunit localizes to the membrane, recruiting the p67^phox^ (activator subunit) and p40^phox^ to the complex. Further, the activation of Nox2 is mediated through an obligatory small molecular weight GTPase, Ras-related C3 botulinum toxin substrate 1 (Rac1), which interacts initially with Nox2 and subsequently with p67phox [20,24]. Rac1 belongs to the Ras superfamily of GTP binding proteins, and its activity is manipulated at various levels due to the inherent and external factors orchestrating its regulation [18,25]. The inherent stipulations are that these are small molecular switches alternating between active and inactive states.

## 4. Rac1

Rac1 promotes ROS generation that participates in the regulation of complex cell biological processes such as transcription factor activation, proliferation, transformation, immune response and apoptosis [26,27,28]. The small GTPases are molecular switches regulating almost every facet of cell biology, and one of the mechanisms involves modulating the gene expression of several transcription factors [29]. Rac1 also activates transcription factors, including nuclear transcription factor B (NF-*k*B) and activator protein 1 (Ap1) [28,30]. Typically, Rac1 is associated with the modulation of actin cytoskeleton, inducing actin polymerization at the membrane to internalize the microorganism and elicit phagocytosis [31,32,33]. However, it is also an integral part of the Nox2 holoenzyme, and thus is also important in regulating ROS levels, and the associated intracellular oxidative stress. 

The regulation and action of Rac1 are not limited to its active and inactive state, but rather extends to its distribution at various sub-cellular spaces where it interacts with different effectors eliciting diverse biological responses. Rac1 cycles between the cytoplasm and the plasma membrane to regulate cellular processes. The cytoplasmic Rac1 is hydrophilic, lacking a transmembrane domain and is rendered hydrophobic by its post-translational modification (e.g., lipidation, prenylation) that drives it to the plasma membrane [18,31]. 

The mitochondrial localization of Rac1 is one of the most recent and novel finding that is gaining scientific interest; Rac1 in the mitochondria elicits varied response in different cell types; the same process could be cytoprotective in one cell type, and toxic in the others. Rac1 interacts with anti-apoptotic mitochondrial outer membrane protein Bcl2, and these interactions play a significant role in determining the fate of a cell [34]. In lymphoma cells, overexpression of Bcl2 inhibits Rac1-mediated apoptosis [34], but in pheochromocytoma-12 cells, these interactions are considered to be protective [35]. A critical regulatory role for Rac1 in the onset of pulmonary fibrosis is considered due to increased H_2_O_2_ production in alveolar macrophages, and Cys-189 of Rac1 is necessary for its mitochondrial import [36]. In diabetic retinopathy, inhibition of Rac1 activity protects retinal endothelial cells from hyperglycemia-induced mitochondrial damage and accelerated apoptosis [15].

Nuclear localization of Rac1 is considered to be cell cycle-dependent; it regulates the centrosome separation and mitotic entry [37]. Nuclear Rac1 also interacts with transcription factors such as STAT3 and NF-*k*B [30]. Several mechanisms have been suggested for nuclear translocation (or import) of Rac1, including post-translational modifications; for example phosphorylation of Rac1 at Thr-108 by extracellular-regulated kinase is considered to be necessary for Rac1 nuclear translocation and accumulation [38].

## 5. The Role of Rac1 in Diabetic Retinopathy

Rac1 plays a critical role in the pathophysiology of diabetic retinopathy by activating Nox2, which leads to excessive free radical generation. As mentioned above, in the pathogenesis of diabetic retinopathy, increased cytosolic ROS produced by Nox2 is an early event, which initiates a cascade of events that elicit mitochondrial damage and capillary cell apoptosis [15,21,39,40]. Activation of Rac1-Nox2-ROS also results in multiple downstream effects, and ROS-mediated alterations of many cellular processes lead to the pathological development and disease [15,41,42]. Experimental models have shown that ROS generated via Nox2 activation precedes mitochondrial damage. Once the mitochondria are damaged, the caspase signaling pathway is activated, resulting in cell apoptosis and the diabetic retinopathy pathology [15,43]. 

Rac1 also activates stress kinases such as p38MAPK, and in addition to their role in mitochondrial damage, stress kinases alter several facets of the retinal physiology that contribute to the pathological development of diabetic retinopathy including alteration in the tight junctions, breakdown of blood retinal barrier and activation of matrix metalloproteinases (MMPs) [44,45,46,47].

As detailed above, Rac1 is pro-apoptotic in the initial stages of diabetic retinopathy, however, Rac1 activation is also associated with aberrant retinal neovascularization; animals models of retinal vein occlusion have demonstrated beneficial effects of silencing Rac1 on retinal neovascularization including choroidal neovascularization [48,49], suggesting it also has a role in the advanced stages of diabetic retinopathy.

## 6. Regulation of Rac1 in Diabetic Retinopathy

The gene expression and activity of a protein is a remarkably complex and regulated process. The complexity lies in the organization of the genetic material and the diversity of players/processes that regulate it. Primarily, the protein levels and its activity are regulated at transcriptional and post-translational level [50,51]. However, in addition, small GTPases exist in alternating GTP-bound active and GDP-bound inactive states, and GTP- and GDP- bound states are regulated by two distinct classes of proteins. While guanine nucleotide exchange factors (GEFs) facilitate GTP binding by releasing the bound GDP, GTPase activating proteins (GAPs) inactivate Rac1 by hydrolyzing the bound GTP. In addition, guanine nucleotide dissociation inhibitors (GDIs) help keep Rac1 in an inactive state by sequestering it away from GEFs [29,39,52]. In mammals, around 70 GAPs and 80 GEFs have been known to regulate the small GTPases, of which over 30 GEFs and several GAPs have been known to regulate Rac1 alone [29]. 

The following sections discuss how Rac1 regulation is altered in diabetes, and its role in the development of diabetic retinopathy.

### 6.1. Functional Regulation of Rac1

#### 6.1.1. Guanine Nucleotide Exchange Factors (GEFs)

Rac1, like most other small G-proteins, is activated by GEFs that facilitate GTP binding by releasing bound GDP [52]. In diabetes, GEF, T cell lymphoma invasion and metastasis (Tiam1), is shown to be critical in orchestrating Rac1 activation; activation of Tiam1-Rac1-Nox2 signaling axis, an early event in the pathogenesis of diabetes, leads to mitochondrial damage and accelerated capillary cell apoptosis. Inhibition of Tiam1 by its specific inhibitor NSC23766, markedly attenuates Rac1 activation, and protects mitochondrial damage and acceleration of capillary cell apoptosis [15].

The same G-protein can also be modulated by multiple GEFs, and Vav2 is considered as another important GEF for Rac1 activation [53,54]; our recent work has implicated Vav2 in the activation of Rac1-Nox2 signaling in diabetic retinopathy. We have shown that the inhibition of Vav2 by its pharmacological inhibitor EHop, in addition to regulating diabetes-induced activation of Rac1-Nox2, also prevents mitochondrial damage, vascular leakage and capillary cell apoptosis. Furthermore, administration of this inhibitor, soon after establishment of diabetes in mice, also inhibits the development of retinopathy [55]. 

Another GEF, Son of sevenless homolog 1 (Sos1), is also implicated in the regulation of Rac1 activation in diabetic milieu. The activity of Sos1 is regulated by 66kDa proto-oncogene Src homologous-collagen homologue (p66Shc) adaptor protein [26,56]. The expression of p66Shc in the human retinal endothelial cells is upregulated in hyperglycemic milieu, and its overexpression displaces Sos1 protein from Grb2, leading to Rac1 activation. P66Shc-mediated activation of Rac1 is facilitated by decreased binding of Sos1 with the growth factor receptor-bound protein 2 (Grb2) [57]. Thus, these pathways seem to be critical in regulating significant downstream signals implicated in the development of diabetic retinopathy.

#### 6.1.2. Guanine Nucleotide Dissociation Inhibitors

**Figure 1:**
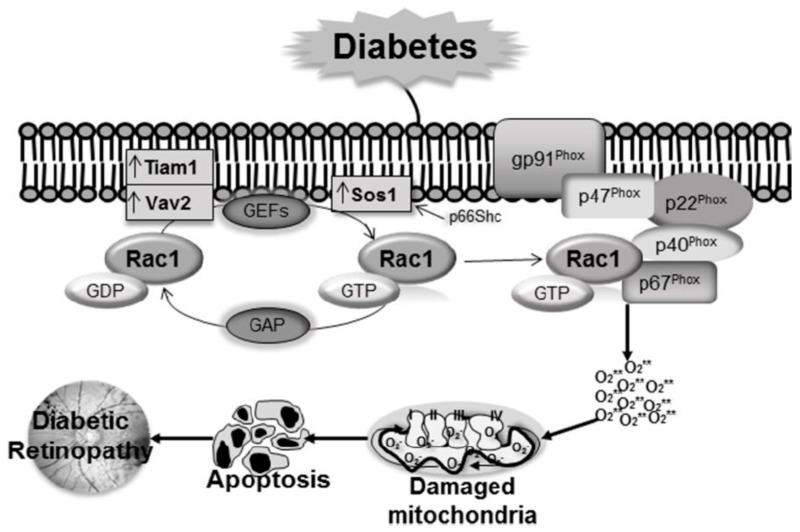
Rac1 activation is regulated by GEFs and GAP. In diabetes Tiam1 and Vav2 are upregulated, and these GEFs activate Rac1 leading to increased Rac1-Nox2-ROS signaling pathway. Furthermore, increase in p66Shc displaces Sos1 from Grb2, leading to Rac1 activation. Hyperglycemia also increases prenylation enzyme FNTA and decreases GDI, that further helps Rac1 translocation to the membrane for Nox2 holoenzyme assembly. Increased ROS production by Nox2 augments mitochondrial damage, leading to capillary cell apoptosis and the development of diabetic retinopathy

Though, GEFs are important regulators of Rac1, additional regulatory control is also rendered by their association with GDIs. In addition to keeping Rac1 away from GEFs, GDIs inhibit their dissociation from the bound GDP, and maintain the small GTPases in the cytosolic compartment [52]. The dissociation of the small GTPases from the GDIs, followed by their post-translational modification, are essential for these GTPases to target the plasma membrane [58]. Experimental models of diabetic retinopathy have demonstrated that in hyperglycemic milieu, while the levels of the prenylating enzyme, farnesyltransferase (FNTA) and GEF Vav2 are increased in the retinal vasculature, that of GDI are decreased, suggesting the importance of GDIs in diabetic retinopathy (Figure 1) [55]. 

#### 6.1.3. Post-Translational Modifications

As detailed above, Rac1 shuttles between the cytoplasm and plasma membrane, and a critical balance is maintained under normal physiological condition. Posttranslational modification of Rac1 is important to drive Rac1 to the plasma membrane; geranylgeranylation and palmitoylation are some of the major post-translational modifications [31,59]. The lipid raft localization of the Rac1 is its major signaling site, and palmitoylation of Rac1 drives it to the lipid raft region of the plasma membrane. However, Rac1 palmitoylation can only be accomplished if it is prenylated and has an intact PBR (C-terminal polybasic region) region [31]. Further, the PBR region helps clustering of the Rac1 at the plasma membrane, increasing protein-protein interaction [60,61]. In the pathogenesis of diabetic retinopathy, the Rac1-Nox2 signaling pathway is activated in the retina leading to oxidative stress, suggesting that Rac1 must be post-translationally modified to be more hydrophobic. In this context, palmitoylation of Rac1 is observed as a prerequisite for its plasma membrane targeting in high glucose condition, and 2-bromopalmitate (2-BP), and inhibitor of palmitoylaton, attenuates the Rac1-Nox2 signaling pathway in retinal endothelial cells [15,59,62]. Furthermore, the prenylation enzyme is increased in hyperglycemic condition, and inhibition of FNTA prevents the activation of Rac1-Nox2-ROS signaling, suggesting prenylation is also an important posttranslational modification for Rac1 activation and the development and progression of diabetic retinopathy [15,63]. Although not examined in the context of the onset of diabetic retinopathy, Rac1 functions have also been shown to be regulated by RNA splicing and other post-translational modifications such as ubiquitination, adenylation, phosphorylation and SUMOylation [64,65], and mutations of Rho GTPases (e.g., Cdc42, Rho and Rac1) have also been reported in pathological states, including immonodeficiency and cancer [64].

### 6.2. Transcriptional Regulation and Epigenetic Modifications

Diabetic environment alters the expression of several genes associated with the development and progression of retinopathy. Gene expression can also be influenced by external factors and disease state, without altering the DNA sequence, and these epigenetics changes define the dynamic and intricate regulatory and functional interactions between DNA, RNA, and protein, ultimately resulting in phenotypes [66,67,68,69]. These modifications can be transmitted to the daughter cells- thus epigenetic modifications can be considered as ‘inheritance, which is not mediated via DNA sequence of genes [70]. However, depending on the regulation of external factors and life style, these epigenetic changes can also be erased/reversed, which make them good therapeutic targets for chronic diseases [71]. Modification of histones, e.g., lysine or arginine residues, methylation of cytosine and noncoding RNAs are some of the major epigenetic modifications. The type of histone modification, e.g., methylation and acetylation, and also the site of methylation, determine whether histone modifications close or open the chromatin structure to regulate transcription factor binding [72]. Interestingly, DNA methylation and histone modifications can also influence each other; histone methylation can help to direct DNA methylation patterns, and DNA methylation may serve as a template for rebuilding histone modification patterns [73].

One of the most widely studied epigenetic modifications is the methylation of cytosine bases. In mammals, methylation is restricted to cytosine residues and mainly encountered in cytosine-guanine dinucleotides (CpG) [74]. Although CpG is usually underrepresented throughout the genome, stretches of 0.3–3 kb (CpG islands) are generally present in the promoter region of a gene [75,76], and their methylation is associated with gene silencing. The addition of methyl group to cytosine is mediated by a family of enzymes, the DNA methyltransferases (Dnmts). Among this family, while Dnmt3a and Dnmt3b are associated with the establishment of *de novo* DNA methylation patterns, Dnmt1 is responsible for the maintenance of established DNA methylation patterns [77]. DNA methylation is a dynamic process, and to maintain proper DNA methylation status, DNA demethylation can be reversed by the conversion of methylcytosine to hydroxymethylcytosine (5hmC) [78]. The demethylation process can either be passive or active, or a combination of both. Passive DNA demethylation is usually on newly synthesized DNA strands, and occurs during replication rounds, and active DNA demethylation is mediated via the sequential modification by the ten-eleven translocation (TET) family of enzymes; 5mC can be converted to 5hmC, 5hmC to 5-formylcytosine (5fC), and 5fC to 5-carboxylcytosine [79]. Among these, the conversion of 5mC to 5hmC is the most common hydroxymethylation. In diabetes, enzyme activities of both Dnmts and Tets are increased in the retina and its vasculature, and among these two families of enzymes, Dnmt1 and Tet2 are the only respective isoforms that are upregulated [80,81,82]. 

**Figure 2:**
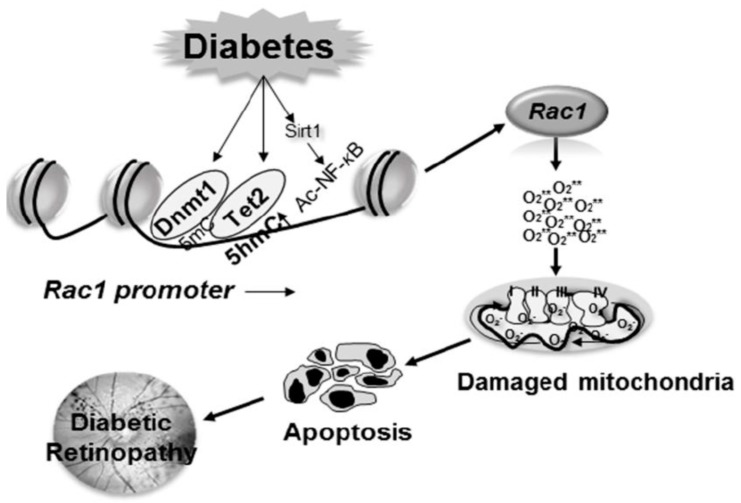
Epigenetic modifications of *Rac1* promoter increase its gene transcription. Activation of Dnmts in diabetes increases methylation of *Rac1* promoter, but concomitant increased binding of the hydroxymethylating enzyme Tet2, 5mC is hydroxymethylated, opening up the chromatin for the binding of the transcription factors. In addition, due to upregulation of SUV39H1, increase in H3K9me3 levels at the promoter further helps the recruitment of Dnmts and the methylation-hydroxymethylation process. Diabetes also inhibits Sirt1, which allows NF-*k*B to be acetylated, and facilitating the binding of the acetylated NF-*k*B at the *Rac1* promoter, further helping in *Rac1* transcription.

*Rac1* transcription is mediated by many transcriptional factors including NF-*k*B and Sp1 [43]. In the pathogenesis of diabetic retinopathy, gene transcripts of *Rac1* are increased in the retina and its vasculature. In diabetes, the binding of both Dnmt1 and Tet2 is increased at its promoter, suggesting an active methylation-hydroxymethylation process of the *Rac1* promoter. 5mC formed by increased Dnmt1 binding is immediately hydroxymethylated by Tet2, and this interplay between two opposing enzymes leaves the promoter hypomethylated. The hypomethylated promoter region allows the transcription factor to bind, ultimately resulting in increased *Rac1* transcription [80]. In addition to regulating the DNA methylation status of *Rac1* promoter, inhibition of Dnmts/Tets prevents NF-*k*B binding. NF-*k*B is also activated in the retina in diabetes, and acetylation of its p65 subunit plays an important role in regulating NF-*k*B-dependent transcription [83,84]. In diabetes, due to hypermethylation of promoter DNA, Sirtuin 1 (*Sirt1*), a NAD-dependent deacetylase, is also inhibited [43,85]. Inhibition of Sirt1 results in impaired deacetylation mechanism, and increases the binding of acetylated NF-*k*B at the promoter, further facilitating *Rac1* transcription (Figure 2).

As mentioned above, many epigenetic modifications are interrelated; DNA methylation can alter H3K9 methylation, and histone modifications can regulate Dnmt1 recruitment to promoter CpG sites [86,87]. Recent studies from our laboratory have shown that due to increased binding of methyltransferase Suv39H1 at the *Rac1* promoter in diabetes, H3K9me3 levels are significantly elevated, and this assists in the recruitment of the DNA methylation machinery, altering the DNA methylation status of *Rac1* and increasing its expression [88].

## 7. Therapeutic Targets

The above discussion clearly shows Rac1 as an important participant in the development of diabetic retinopathy; the diabetic environment functionally activates Rac1 in the retina and its vasculature, and alterations in the epigenetic machinery influence its transcriptional activation, making Rac1 as an excellent target for therapeutical intervention. As Rac1 is regulated via several processes, it provides an opportunity to identify the critical processes and mediators essential for its activation and, thus, their regulation to inhibit Rac1 activation. Several pharmacological approaches have been used to target these and inhibit Rac1 activation, and one of the most obvious among these is targeting the GEFs. Administration of inhibitors of Tiam1 and Vav2, inhibit Rac1-Nox2 signaling in diabetic animal models, and prevent the development of diabetic retinopathy [15,55]. Pharmacological and molecular inhibitors of posttranslational modifications, and the enzymes associated with such modification provide another opportunity to regulate Rac1 activation [42,89].

Rac1-Nox2 is also regulated by the inhibitors of 3-hydroxy-3-methylglutaryl coenzyme A (HMG-CoA); simvastatin has been shown to decrease Rac1 activation and increase in nitric oxide in spontaneously hypertensive rats [90]. Although human trials investigating the effect of statins on Rac1 are very limited, they have shown positive effects on blood vessels [91,92,93]. A recent population-based cohort study conducted in Taiwan has shown a decreased risk of diabetic retinopathy, and its progression to vision-threatening diabetic retinopathy in Taiwanese patients receiving statin therapy [94]; additional studies are needed to ascertain the link between statins and diabetic retinopathy. Furthermore, since nitric oxide can also activate Rac1 [95], several nitric oxide releasing molecules have been analyzed for their protective effect against oxidative stress, and VP10/39 (caffeic acid phenethyl ester derivative) has been shown to protect retinal pigment epithelial cells against oxidative stress [96]; however, the effect of such molecules on diabetic retinopathy remains to be investigated.

As stated above, pharmacological investigations involving Rac1 inhibitors have suggested Tiam1 (NSC23766), Vav2 (Ehop-016) and SoS1 as GEFs involved in sustained activation of Rac1 in the retina in diabetes (Figure 1). It is important to note that other pathological conditions including lipotoxicity (ceramide-mediated) or chronic inflammation (proinflammatory cytokines, such as IL-1β) could also mediate their cytotoxic effects *via* activation of Tiam1/Vav2-Rac1-Nox2 pathway, thus suggesting that this signaling axis might be “druggable” to prevent/halt metabolic defects. However, some aspects remain speculative and needs to be confirmed experimentally including potential cross-talk and/or inter-dependence, if any, between Tiam1, Vav2 and SoS1 in mediating their effects on Rac1 activation, and potential regulatory mechanisms upstream to the activation of these GEFs. Furthermore, it is critical to understand the subcellular compartmentalization of these GEFs and Rac1 in diabetes and/or glucolipotoxic conditions that could potentially lead to mistargeting/mislocalization of Rac1 (e.g., translocation to nuclear compartment), culminating in cellular dysfunction and loss. We recognize that Rap1, which represents one of the components of the membranous core of Nox2, could also contribute significantly in the subcellular-localization (e.g., plasma membrane) of GEFs in the steps leading to Rac1 activation and optimal assembly of the Nox2 complex, possibly providing additional therapeutic targets. 

Since *Rac1* is also transcriptionally activated in diabetes, and epigenetic modifications modulate its transcriptional activation, targeting the enzymes responsible for such modifications provides another opportunity to regulate Rac1 activation. As mentioned above, increased Dnmt binding, through forms more 5mC, but simultaneous activation of Tet2, hydroxymethylates *Rac1* promoter. This makes Dnmts as a good target to inhibit Dnmt binding- hydroxymethylation. Over the past decade, many epigenetic-modifying agents have been developed, and are being employed in the clinical management of patients with malignancies [97,98]. Demethylating agents, azacytidine and decitabine, are being used for the treatment of myelodysplastic syndromes, but these agents carry a burden of high toxicity [99,100]. Some of the synthetic DNA methylation inhibitors such as hydralazine and procainamide are now being used in clinical trials for tumors, and these inhibitors have less adverse side effects than azacytidine and decitabine [101]. In addition, since histone methylation also plays an important role, inhibitors of methyl transferases also carry a therapeutic advantage [102,103]. Downregulation of deacetylase Sirt1 allows NF-*k*B to remain acetylated, suggesting the possibility of increasing Sirt1 activity could refrain NF-*k*B for being in acetylated state to bind at the promoter. This raises the possibility of use of dietary flavonoids and polyphenols such as resveratrol and catechins, which may upregulate Sirt1, and also directly inhibit Dnmts [104,105]. Although dietary flavonoids and polyphenols seem to be attractive therapeutic models, due to their varying pharmacokinetic profiles, their physiological relevance with supporting clinical findings needs to be investigated. Furthermore, modulation of epigenetic enzymes has many limitations including silencing/activation of other genes, and less than desirable drug specificity [106]. Thus, the selection and rational prioritization of epigenetic agents are important for identifying the most appropriate agents for patients in clinical practice. Further, the blood-retinal barrier poses another challenge for these drugs to reach to the retina, which should always be considered. 

Computational systems biology is gaining the interest of the scientific community for identifying drug targets, and has potential considering the complex pathology of diabetic retinopathy. The analysis of transcriptomics data (retrieved from Gene Expression Omnibus Dataset repository datasets) has identified some genes and biological pathways related with inflammation, fibrosis and G protein-coupled receptors involved in the development of this blinding complication of diabetes [107]. However, additional analysis using different data sources are warranted to gain interesting insights/leads into the molecular pathways implicated in the pathogenesis of diabetic retinopathy.

Retina has an organized blood-retina barrier [108], which makes topical delivery of drugs to the posterior segment of the eye a challenging task [109]. However, the use of nanocarriers to formulate sustained delivery of the compounds [110], to prevent functional and/or transcriptional activation of Rac1 remains an attractive avenue.

In conclusion, as detailed above, in the pathogenesis of diabetic retinopathy once mtDNA is damaged, due to dysfunctional electron transport chain, the vicious cycle of ROS continues to self-propagate, but activation of Rac1-Nox2 precedes mitochondrial damage. Thus, inhibition of Rac1 activation, an early event in the pathogenesis of diabetic retinopathy, will halt the vicious cycle of ROS accumulation, and ameliorate further progression of the disease. Furthermore, Rac1 is also associated with aberrant retinal neovascularization, and its inhibition during the advanced stages of retinopathy would also slow down neovascularization, a hallmark of proliferative diabetic retinopathy [48]. Optimistically, efforts are being put into developing strategies to regulate Rac1 activation for the treatment of other chronic diseases, and Dnmt inhibitors are also being used in clinical trials for tumors. The delivery of these drugs, along with maintenance of sensible glycemic control, holds promise to retard the development/progression of retinopathy, and prevent vision loss in diabetic patients.
